# 35. To kill or cure? The diagnostic challenge in a patient with cerebral lupus

**DOI:** 10.1093/rap/rky033.026

**Published:** 2018-09-20

**Authors:** Mark D J Gibson

**Affiliations:** Department of Neurology, Cambridge University Hospitals, Cambridge, UNITED KINGDOM

**Introduction:** Lupus patients presenting with neurology symptoms continues to represent a diagnostic challenge for clinicians as the neurology may be attributable manifestation of their disease or as a result of immunosuppression. Differentiating disease flares from infection in such patients is crucial given the diametrically opposed treatment strategies however no definitive tests to distinguish flare from infection are currently widely available. This case highlights the diagnostic challenge and treatment dilemmas patients with neurological manifestations of systemic lupus erythematosus (SLE) can present.

**Case description:** A 67 year old female with a history of SLE was transferred to the neurological unit of this hospital with fluctuating confusion and fevers. She presented to her local hospital nine days previously with a three day history of confusion, lethargy and fevers. On assessement in her local emergency department she was febrile (temperature 38.1) with an elevated CRP of 68 mg/dL. She received a diagnosis of urinary sepsis and was commenced on co-amoxiclav. She continued to be febrile. An MRI scan of the head revealed multiple lacunar infarcts across several vascular territories suggestive of embolic infarcts. A transthoracic echocardiogram did not note any valvular vegetations but was significant for moderate mitral regurgitation. A lumbar puncture revealed a normal white cell count of < 2, protein 1.47, glucose 1.8, paired serum glucose 5.1. The patient was transferred for further assessment and investigation. The diagnosis of SLE was made six years prior to admission, at the age of 61, following a presentation with renal and cardiac failure. Her antibody profile at that time revealed positive titres for ANA, anti-Ro and anti-dsDNA. Hypocomplementaemia was noted. A renal biopsy was significant for class IIIa lupus nephritis. During her inpatient stay she suffered three cardiac arrests resulting in a prolonged intensive care admission and the development of a critical care myopathy. She was given a diagnosis of lupus cardiomyopathy. She was commenced on high dose steroids and mycophenolate mofetil 500 mg twice daily which was subsequently increased to 2 g daily with resulting good disease control. Hydroxychloroquine was commenced but discontinued due to a rash. In December 2016, one year prior to admission, during a period of mycophenolate withdrawal the patient suffered an ischaemic stroke. Four months prior to admission the patient suffered a deep vein thrombosis of the lower limb and received a three month course of rivaroxaban. On arrival at this hospital the patient was drowsy with a GCS score of 12. Examination revealed a pan-systolic murmur, no peripheral stigmata of infective endocarditis, an erythematous facial rash sparing the nano-labial folds was noted, there was no livedo reticularis. Plantar reflexes were extensor bilaterally. Over the subsequent 24 hours the patient’s GCS fluctuated between 9 and 12. Blood tests revealed a mildly elevated ESR of 38 mm/hour with a significantly elevated CRP of 108 mg/dL. A serum procalcitonin was not elevated. Antibody titres for dsDNA were normal. Complement levels (C3 and C4) were normal. A lupus anticoagulant screen revealed a mildly positive anti-beta-2-glycoprotein-1 IgG with normal titres anti-beta-2-glycoprotein-1 IgM, anticardiolipin IgG and IgM and a negative lupus anticoagulant. Therapeutic low molecular weight heparin was commenced. Repeat MRI confirmed the presence new embolic ischaemic lacunar strokes. A repeat lumbar puncture revealed an elevated lymphocyte of 28, a polymorph count of 4, protein 1.08 g/dL, CSF glucose 2.1 (paired serum glucose 6.2). Antibiotics were discontinued and blood cultures taken when the patient was febrile. A transoesophageal echocardiogram was requested to assess for the presence of vegetations. A digital subtraction angiogram was requested to assess the intracranial vasculature. The patient’s GCS deteriorated and they were transferred to the neurological intensive care unit. They were commenced on ceftriaxone. Despite supportive therapy on the ICU on the tenth day of admission, neuroimaging confirmed a devastating intracranial haemorrhage. The patient died on the eleventh day of admission.

**Discussion:** At the time of the patient's death the differential diagnosis of the patient's presentation included: cerebral lupus, anti-phospholipid syndrome, central nervous system vasculitis, infective endocarditis, Libman-Sacks endocarditis and an infective meningitis. No immunosuppression was administered whilst the aetiology of the patient's presentation was investigated. A hospital post mortem was performed to further ascertain the cause of death. Post-mortem examination revealed a florid necrotising vasculitis throughout the brain and its leptomeningeal coverings. This was associated with multiple acute and subacute infarcts and ultimately fatal intracerebral haemorrhage and haemocephalus. There is no evidence of infectious disease. Examination of the heart revealed no valvular vegetations. Cerebral lupus vasculitis is a rare manifestation of SLE (7-12% SLE post mortem studies) and is associated with a high mortality (∼67%). Antiphospholipid syndrome, cerebral lupus vasculopathy, infective aetiologies and Libman-Sacks endocarditis are more common causes of neurological presentations in SLE patients.

**Key Learning Points:** A florid necrotising cerebral lupus vasculitis can occur despite normal anti-dsDNA titres and complement levels (traditional markers of lupus activity). Despite the high C-reactive peptide and modest erythrocyte sedimentation rate values observed in this patient, no evidence of an infective process was found at post mortem suggesting the elevated CRP was attributable to cerebral vasculitis

**Disclosure: M.D.J. Gibson:** None.

